# Combining gene expression data from different generations of oligonucleotide arrays

**DOI:** 10.1186/1471-2105-5-159

**Published:** 2004-10-25

**Authors:** Kyu-Baek Hwang, Sek Won Kong, Steve A Greenberg, Peter J Park

**Affiliations:** 1 School of Computer Science and Engineering, Seoul National University, Seoul 151-742, Korea; 2 Molecular Medicine, Beth Israel Deaconess Medical Center, 330 Brookline Ave, Boston, MA 02215, USA; 3 Bauer Center for Genomics Research, Harvard University, 7 Divinity Ave, Cambridge, MA 02138, USA; 4 Department of Neurology, Brigham and Women's Hospital, 75 Francis Street, Boston, MA 02115, USA; 5 Children's Hospital Informatics Program, 300 Longwood Ave, Boston, MA 02115, USA; 6 Harvard-Partners Center for Genetics and Genomics, 77 Avenue Louis Pasteur, Boston, MA 02115, USA

## Abstract

**Background:**

One of the important challenges in microarray analysis is to take full advantage of previously accumulated data, both from one's own laboratory and from public repositories. Through a comparative analysis on a variety of datasets, a more comprehensive view of the underlying mechanism or structure can be obtained. However, as we discover in this work, continual changes in genomic sequence annotations and probe design criteria make it difficult to compare gene expression data even from different generations of the same microarray platform.

**Results:**

We first describe the extent of discordance between the results derived from two generations of Affymetrix oligonucleotide arrays, as revealed in cluster analysis and in identification of differentially expressed genes. We then propose a method for increasing comparability. The dataset we use consists of a set of 14 human muscle biopsy samples from patients with inflammatory myopathies that were hybridized on both HG-U95Av2 and HG-U133A human arrays. We find that the use of the probe set matching table for comparative analysis provided by Affymetrix produces better results than matching by UniGene or LocusLink identifiers but still remains inadequate. Rescaling of expression values for each gene across samples and data filtering by expression values enhance comparability but only for few specific analyses. As a generic method for improving comparability, we select a subset of probes with overlapping sequence segments in the two array types and recalculate expression values based only on the selected probes. We show that this filtering of probes significantly improves the comparability while retaining a sufficient number of probe sets for further analysis.

**Conclusions:**

Compatibility between high-density oligonucleotide arrays is significantly affected by probe-level sequence information. With a careful filtering of the probes based on their sequence overlaps, data from different generations of microarrays can be combined more effectively.

## Background

By providing a genome-wide view of gene expression, microarrays have become a common exploratory tool in many areas of biological and clinical studies [1-3]. While there are several different microarray platforms, photolithographically synthesized oligonucleotide arrays from Affymetrix have become one of the principal technologies. These arrays feature multiple 25-mer probes (a "probe set") for each gene, with their measurements summarized into a single number for the estimated expression level of that gene. Because of the important role played by this technology, many methodological studies have focused on improving the extraction of information from these arrays, from image analysis and the proper role of perfect and mismatch probes to distributional properties of the measurements and optimal statistical tests for differential expression [4,5].

Large-scale gene expression data often contain a large amount of noise from various experimental factors. Fortunately, in most cases, the technical variability is relatively small compared to the biological one and its effect can be minimized by using a sufficient number of replicates [6-8]. However, the high cost of microarray experiments often prevents gathering of enough samples for a reliable analysis in a single laboratory. In such cases, employing existing microarray datasets from other studies can be an efficient way of improving the reliability of a study. Moreover, as the number of publicly available datasets grows rapidly on public data depositories (e.g., Gene Expression Omnibus [9]; Stanford Microarray Database [10]; ArrayExpress at EBI [11]), it is clear that these datasets should be combined to generate a more comprehensive understanding of underlying biology.

Several issues have made this process difficult so far. First, different datasets have been processed using different procedures due to a lack of uniform standards, e.g., for background correction, normalization, and calculation of expression values. This makes it difficult to compare them directly. Raw data files are generally unavailable and, even if they are, reprocessing them requires substantial effort. Second, we have lacked datasets with enough controls and replicates, performed under a proper experimental design and with adequate annotations, in order to make proper comparisons. Third, possibly the most troublesome, the experiments have been performed on many different platforms, with significant differences among them. Even within a single platform, technological and algorithmic advances as well as the evolving annotations of the genomes have resulted in succeeding generations of arrays with substantial modification from one generation to the next. Until now, several studies have found varying degrees of disagreement between platforms, sometimes with large discrepancies that call into the question the reliability of certain conclusions reached in microarray studies [12-19]. A comparison of two Affymetrix arrays, HuGeneFL and HG-U95A, was made previously, but only with the conclusion that the reproducibility is high when the two probe sets share many exact probes and that it is low when they do not [20].

**Figure 1:**
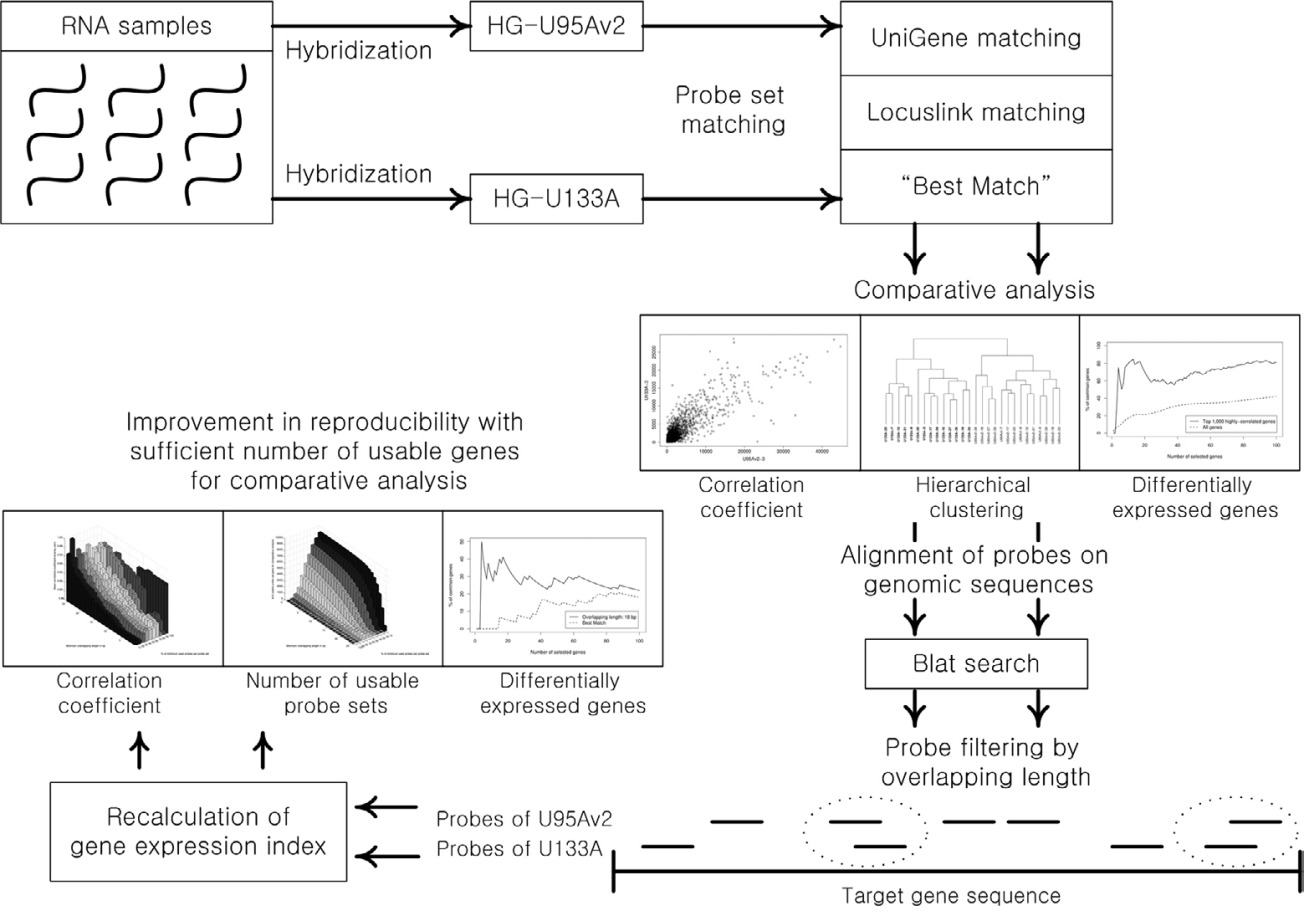
A schematic view of the procedure. The same RNA was hybridized on both HG-U95Av2 and HG-U133A arrays, for 14 samples. Three methods for matching the probes were considered, but the two datasets gave highly inconsistent results in cluster analysis and identification of differentially expressed genes. To improve the comparability in general, probe-level sequence information was exploited. All 25-mer probes were aligned to human genome sequences by BLAT and then filtered based on the length of their overlap with the probes on the other array. New expression indices were calculated using only the selected probes, and this results in higher reproducibility.

In this work, we carry out a thorough examination of the comparability between the two generations of Affymetrix human GeneChip arrays, HG-U95Av2 and HG-U133A, both of which have been used extensively for studying human gene expression patterns. We then propose a method for enhancing their comparability. The analysis we carry out is made possible by a dataset consisting of the same tissue samples hybridized on both platforms. The procedure is illustrated in Figure 1. Using our replicate dataset, we first examine the effectiveness of three schemes for matching the probe sets across different arrays. We then quantify the surprising amount of difference in analysis results between the platforms, as revealed in correlation analysis, hierarchical clustering, and selection of differentially expressed genes. We find that comparability can be improved by rescaling expression values or data filtering but that these techniques are limited to few specific analyses. As a generic method for comparative analysis, we propose selecting a subset of probes that have sequence overlaps with the probes on the other array and recalculating the expression levels based only on this subset. We demonstrate that this probe filtering significantly improves the reproducibility, without eliminating a significant number of genes from the analysis.

## Results

### Comparison of the methods for probe set matching

The most common method of matching genes in cross-platform studies is to match the UniGene IDs among genes [12,15-18]. One potential problem with this method is that as the UniGene database is updated, some tags are retired and new ones are created, and these may not be tracked correctly unless the same version of UniGene was used to annotate each platform. LocusLink does not suffer from this problem as much and therefore may be preferable in some cases. We tested three methods for matching probe sets between U95Av2 and U133A: UniGene IDs, LocusLink IDs, and Best Match provided by Affymetrix [21]. As shown in Table 1, there are about 9000 unique IDs shared between U95Av2 and U133A in all three cases, with slightly more for the Best Match. The number of probe sets involved is higher for UniGene and LocusLink matching, since there are multiple probe sets corresponding to the same ID in those cases. For Best Match, the sequence mapping is restricted to many-to-one matching.

As a simple way to assess comparability, the Pearson correlation coefficient between each array pair from the same sample was calculated and the 14 correlation coefficients were averaged. The results are summarized in Table 1. UniGene and LocusLink matching give practically identical results. Best Match, on the other hand, shows somewhat higher reproducibility than other matching methods (.870 vs .831–.832). The main reason for the higher reproducibility in Best Match is most likely that more comparable probes are chosen among multiple matches by considering the sequence information. The overall reproducibility, however, is surprisingly low. It has been observed in many replicate studies that expression values from Affymetrix arrays show high reproducibility, typically in the range of >0.98 [20,22,23]. The low correlation coefficient is already an indication that the cross-generation comparison may not be simple. We use the Best Match in the following sections; UniGene or LocusLink matching performs similarly or slightly worse than Best Match.

**Figure 2:**
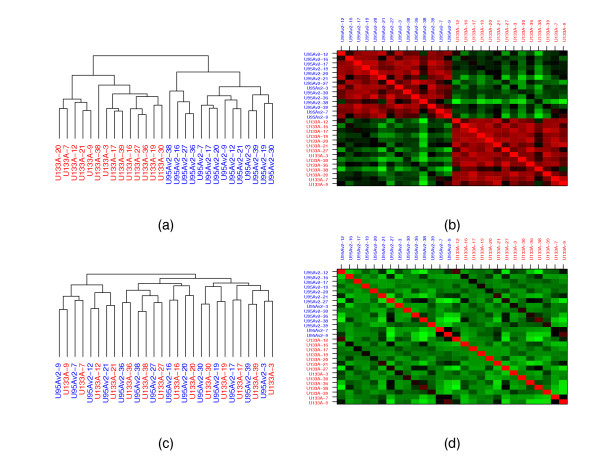
Cluster analysis on the combined dataset from U95Av2 and U133A. (a) The result of hierarchical clustering of 28 samples using the Pearson correlation coefficient as a distance measure. The dendrogram is exactly divided into the two groups representing U95Av2 and U133A, rather than by sample type. (b) The correlation coefficients between every two arrays in the combined dataset. The red (green) color corresponds to a higher (lower) value of correlation coefficient. The bias between U95Av2 and U133A is clearly represented here (upper left and lower right parts of the rectangle). (c) The result of clustering after gene scaling. Each gene expression value was scaled across the samples before combining U95Av2 and U133A datasets. In the dendrogram, the arrays obtained from the same biopsy are now joined together in all cases. (d) The bias between U95Av2 and U133A has clearly disappeared by gene scaling. The dark red diagonals in the upper right and lower left parts denote the high correlation coefficients for the same biopsy hybridized on different arrays.

In a similar study [24], the authors report the average correlation of .81 ± .01 between two different generations of Affymetrix Arabidopsis arrays. But they conclude that this reproducibility is sufficiently high and that the array generations can be compared without further manipulation of the data. However, in our experience, this number is much too low. In the current data set, for instance, the samples in different disease groups give significantly higher correlation coefficients than that. This is clearly demonstrated later in Figure 2(b), where the arrays in the same generation are shown to be more highly correlated than the arrays in the same disease class.

### Exactly matched probes between array generations are highly reproducible

There was a possibility that the lack of high correlation between the two versions was caused by a true inconsistency present in the data, perhaps due to RNA degradation between the times when the hybridizations on the two platforms were performed. To make sure that this was not the case, we investigated the quality of our data by examining the subset of probes which have the exactly same sequences between the array generations.

When we examined about 5% of probes that have the same sequence between U95Av2 and U133A, the mean correlation coefficient of array pairs, calculated by PM intensity, was 0.967 ± 0.007. (A calculation using PM-MM values also gives a very similar result.) This is similar to the conclusion in [20] that the probe sets with exactly the same set of probes have a very high correlation. The high correlation in our dataset confirms that the samples and other experimental factors were nearly identical between the two hybridizations and that any discordant result in comparative analysis is therefore most likely due to the differences in the probe design of the two arrays. When we compare the expression values between Best Match and the exactly matched probes, we can easily see the lack of reproducibility for the Best Match case (See Figure 2 in Additional File 1). It is clear that the probe-level sequence information has a large impact on the relationship between the abundance of transcript and the reported intensity [25] and that the use of probe sequences would be necessary in order to choose a subset of relatively consistent probes between U95Av2 and U133A for enhanced reproducibility.

### Standard probe set matching produces discordant results in analyses

To determine the extent to which the analysis results from the two versions of the arrays agree, we employ the two most frequent tools for exploratory analysis: cluster analysis and identification of differentially expressed genes. For evaluating the compatibility in terms of cluster analysis, we combined the datasets from U95Av2 and U133A by Best Match. Then, the 28 samples were clustered by agglomerative hierarchical clustering method with the Pearson correlation coefficient as the distance measure. Figure 2(a) shows the dendrogram of 28 samples. Unexpectedly, instead of each array pair from the same biopsy specimen clustering together, the two array types form the two main clusters. In other words, the most distinguishing feature of the data is the array version, rather than the actual characteristics of the samples. To examine the reason for this incongruent result, correlation coefficients of all the possible sample pairings of the combined dataset were calculated. Figure 2(b) shows the correlation coefficients as a color map. The two red parts of the map (upper left and lower right) represent the high correlation coefficients among samples from the same array version. Compared to these, the correlation coefficients across U95Av2 and U133A are relatively low (lower left and upper right parts of the map).

Next, we identified differentially expressed genes between the DMs and other myopathies from each dataset (5 vs 9 samples), using the two-sample *t*-test with unequal variances (the Wilcoxon test gives very similar results). If the two generations of arrays were comparable, the lists of differentially expressed genes should contain many overlapping genes. To increase the possibility of overlaps, we filtered out non-expressed genes by deleting those in which more than 75% of the samples received Absent calls in both U95Av2 and U133A arrays. When we examine the list of genes identified in common in the two cases, however, its length is disappointingly small. When we look at the list of length 100 or smaller, the percentage of overlap does not exceed 25%. The plot of the percentage of genes common in both lists as a function of the list size is virtually identical to the dashed line in Figure 7b (A detailed plot is shown in Figure 4 of Supplementary Material). This low overlap indicates that the two array types give highly inconsistent results and brings into question the reliability of the highly ranked genes in either platform. We do note, however, that this result must be interpreted in terms of the sample size and other characteristics of the specific dataset. A low percentage is often partially due to the presence of a large number of genes that are differentially expressed to a similar extent in a particular dataset, in which case a ranking of the genes would be expected to be somewhat unstable.

### Gene scaling and data filtering can enhance comparability in specific situations

**Figure 3:**
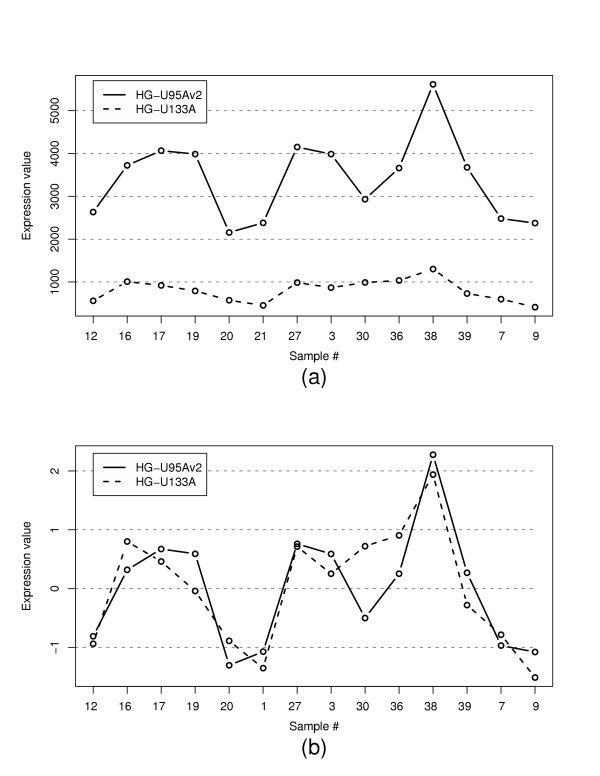
Expression pattern of probe sets for the same gene: 35828_at in U95Av2 and 208978_at in U133A (matched by Affymetrix "Best Match"). (a) The expression patterns before gene scaling. Even though their shapes are somewhat similar, their scales are very different, (b) The expression patterns after gene scaling. After gene scaling, the similarity in the patterns is more clearly visible and these genes have a comparable effect on the clustering of the samples.

To understand the reason for the discordance observed in Figure 2(a), we have examined a large number of probes. The underlying problem, we have discovered, is due to a large number of probe sets that exhibit similar relative expression patterns but at different absolute levels. As an illustration, we plot the expression pattern of one such probe set pair, 35828_at of U95Av2 and 208978_at of U133A, in Figure 3(a). Clearly, although the expression patterns of these genes are similar in terms of a correlation coefficient, their scales are very different. This behavior is not simple to explain, but we believe it may be related to a large amount of cross-hybridization by a subset of badly designed probes in a probe set, especially for U95Av2. That would have the effect of amplifying the overall expression values.

A simple solution to this problem is to scale expression values for each gene across samples, for instance, making the mean to be 0 and the standard deviation to be 1. The effect of this gene scaling on the gene pair from Figure 3(a) is illustrated in Figure 3(b). The similarity in the expression pattern is more clearly visible and the measurements for this gene are now more comparable. While the Pearson correlations for the genes are not impacted by this linear scaling for genes, the correlations do change for the arrays. Figures 2(c) and 2(d) show the effect of gene scaling on the clustering result and the correlation coefficient of sample pairs, respectively. In Figure 2(c), the arrays from each platform corresponding to the same sample are now clustered together in every case. In Figure 2(d), the high correlation among the arrays of same type (shown by red colors in Figure 2(b)) is diminished and the correlation between specimen samples across array types is highlighted (shown by dark red diagonal lines in upper right and lower left areas). For comparing datasets in a cluster analysis, gene scaling appears to work very well.

**Figure 4:**
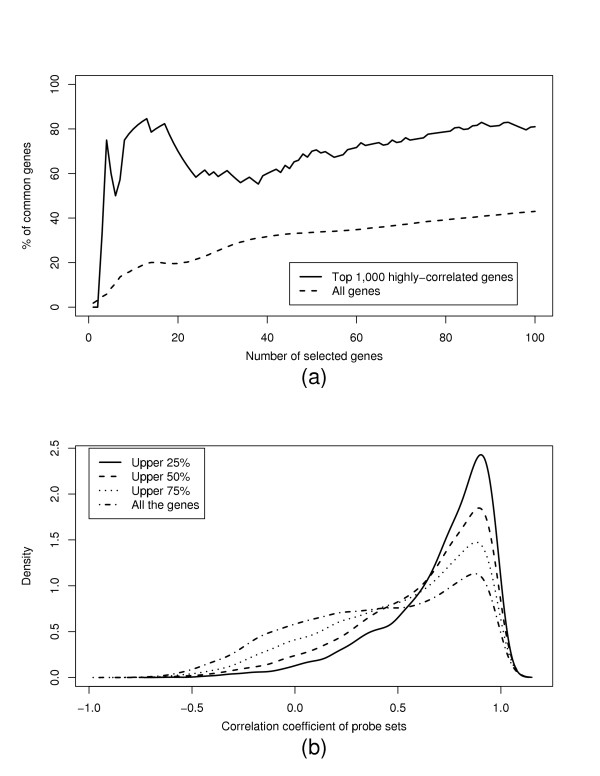
The effect of data filtering on identification of differentially expressed genes and on correlation between array types for the same genes. (a) Percentage of differentially expressed genes common in U95Av2 and U133A datasets. When we considered only the top 1,000 highly correlated genes across U95Av2 and U133A, the overlap between the lists of differentially expressed genes increased dramatically (solid line). For comparison, we show the result without gene selection by correlation (dashed line). For the latter, we subsampled a random gene set of same size repeatedly to eliminate the effect of total size; we also filtered using Present and Absent calls to increase the overlaps. (b) Distribution of the correlation coefficient of probe sets stratified by their mean expression value across U95Av2 and U133A. The density was estimated for upper quartiles using a Gaussian kernel. Filtering by expression values clearly enhances the correlation of probe sets across array types, thus improving the reproducibility in the selection of differentially expressed genes.

While gene scaling was effective in cluster analysis, it is limited to evening the influence of different genes in a global analysis by focusing on their patterns. It does not enhance the comparability, for instance, in terms of identifying differentially expressed genes in most algorithms. For that case, some simple filtering schemes could enhance reproducibility instead. One way is to consider only the genes that exhibit strong correlations between the two versions. To see the impact of this on the selection of differentially expressed genes, we calculated the overlap for the 1,000 genes whose profiles on the two array versions were highly correlated. The result is plotted in Figure 4(a) (solid line). To make sure that the increase in the overlap percentage is not due to the smaller number of genes, we also calculated the overlap for bootstrap samples of same size and averaged the result in Figure 4(a) (dashed line). As expected, data filtering by correlation coefficients greatly improved the comparability, more than doubling the percentage of genes in common. With more datasets such as the one we examine here, it is in theory possible to catalog a comprehensive list of genes that are reproducible across arrays, and use only these genes in subsequent comparative studies. Instead of choosing highly-correlated gene pairs, we can also filter data by expression values. Figure 4(b) shows the distribution of correlation coefficients for genes between the versions stratified by their average expression values. We first note that the distribution for all genes is very wide, with the Pearson correlation coefficient of .426 ± 390, reflecting the poor concordance for the probe set values on the two platforms. With the stratification, it is clear that highly expressed genes tend to give more reproducible expression patterns across the two versions, although there still is a fraction of genes with low or even negative correlation. The disadvantage of this type of filtering is that, as in the filtering by correlation, it inevitably reduces the number of probe sets for the analysis significantly.

### Probe filtering by overlapping length highly improves reproducibility with enough probe sets for comparison

We now describe a more general method for improving comparability by filtering at the probe level, instead of at the probe set level. We have already observed that the probes with exactly the same sequences on the two generations give highly reproducible values (Additional File 1, Figure 2) but that the probe sets do not. This implies that specific probe sequences within the same target region can produce strikingly different results, and suggests that comparability would improve if we select only those probes that have sequence similarities on the two arrays. To carry this out, we mapped the location of all probes using BLAT, as described in Methods. When we select a subset of probes, we mask the rest in the raw data (cel files) and then recompute the expression values using the same algorithm used in MAS 5.0.

**Figure 5:**
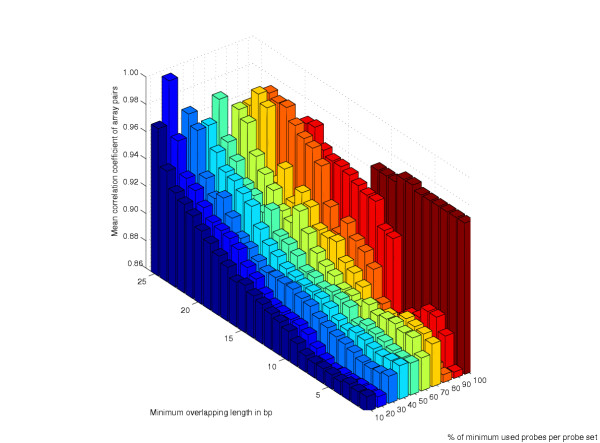
Improvement in the correlation coefficients of array pairs for the same biopsy according to the minimum overlapping length (1 ~ 25 bp) and the percentage of used probes per probe set (10 ~ 100%). The correlation coefficients are the average of 14 arrays pairs. The probes are selected based on sequence overlap, and the probe sets with a sufficient number of such probes are used to recalculate the expression profiles. The correlation is enhanced with increasing number of minimum overlap length and, to a lesser extent, with increasing percentage of probes required for probe set.

An optimal selection scheme requires a balance. On the one hand, we would like to require as large a sequence overlap as possible between the probes to ensure high reproducibility. On the other hand, a stringent restriction means that the number of usable probe sets in an array is reduced and also that each probe set value will be less robust because it is derived from fewer probes. Figure 5 shows the correlation coefficient of array pairs from the same sample according to two criteria: the minimum overlapping length (1 bp ~ 25 bp) and the minimum fraction of used probes per probe set (10% ~ 100%). The latter refers to the fraction for each probe set, e.g., 30% minimum means that at least 4 out of 11 probes for U133A and 5 out of 16 for U95Av2 must satisfy the sequence overlap requirement. If there are too few probes left in a probe set, we discard the probe set as unreliable.

**Figure 6:**
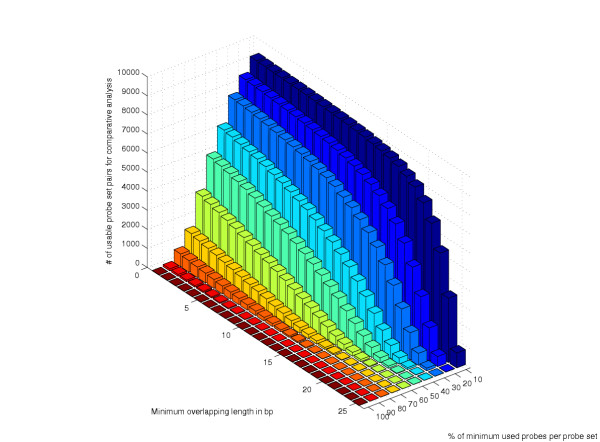
The number of retained probe set pairs for comparative analysis according to the same two criteria as in Figure 5.

In Figure 5, we plot the average of the correlations for the pairs of U95Av2 and U133 chips on which the same sample is hybridized. We see that the average correlation improves substantially with the greater amount of sequence overlap at all ranges. It also improves with the minimum percentage of probes used but only slightly. Figure 6 shows the number of usable probe set pairs according to the same two criteria. It appears, for example, that we can obtain highly comparable results (correlation coefficient > 0.9) with a large number of probe sets (more than 80%) for comparative analysis. For a given value of minimum overlap length, we can also calculate the average number of probes per probe set (See Figure 5 in Supplementary Material) in addition to the number of retained probe sets. With 20 bp minimum overlap, more than 90% of probe sets can be used, with the expression levels calculated from an average of 30% of the original probes per probe set.

**Figure 7:**
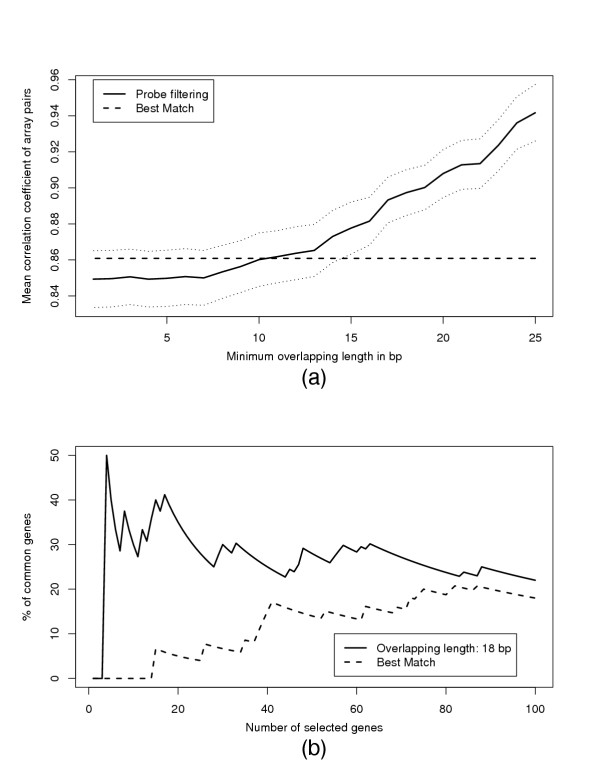
Effect of probe filtering on the comparability between U95Av2 and U133A. (a) We plot the mean correlation coefficient of array pairs from the same biopsy according to the minimum overlapping length used for probe filtering. (standard deviations are drawn as thin dotted line.) Improvement in reproducibility is roughly proportional to the allowed minimum overlapping length. For comparison, the mean correlation coefficient with all 10,507 probe set pairs of Best Match is also drawn (dashed line). For significant enhancement in comparability, the minimum overlapping length should be more than 15 bp. (b) Improvement of reproducibility in the selection of differentially expressed genes (DM vs others). Here, we compare Best Match (10,507 probe sets) with having a minimum overlapping length of 18 bp (9,515 probe sets). The reproducibility was markedly improved by probe filtering, especially for the top ranked genes.

To emphasize the improvement, we again show in Figure 7(a) the increase in the mean correlation coefficient of array pairs, without any criterion on the fraction of used probes per probe set. As a baseline, the mean correlation coefficient of array pairs using Best Match is also represented (dashed line). Enhancement in the mean correlation coefficient of array pairs is roughly proportional to the minimum overlapping length. It appears that the mean correlation coefficient can be worse than in the case of Best Match when the minimum overlapping length is less than 10 bp. It is possibly because such a small overlap constitutes enough dissimilarity as to confer no functional relationship between the probes and instead other good probes that do not have overlaps are thrown away. Based on Figures 5 and 7(a), we suggest that the minimum overlapping length of more than 18 bp is necessary for obtaining significantly improved results in terms of correlation coefficient of array pairs (>0.9).

Next, we show the improvement of comparability in terms of selecting differentially expressed genes. Figure 7(b) shows the percentage of commonly identified differentially expressed genes between U95Av2 data and U133A data when the probes are filtered with minimum overlapping length of 18 bp. The number of usable probe set pairs in this case is more than 9,500. For comparison, the result for the Best Match (10,507 probe set pairs) case is also drawn (dashed line). From Figure 7(b), it is clear that the improvement in comparability is significant, especially when the number of selected genes is small. For example, without the probe filtering, the lists of top 15 genes in the two data sets have no genes in common; with filtering, 30 ~ 50% of the genes are shared. These results demonstrate that the filtered and recomputed data sets are more comparable with only a small reduction in the number of usable probe sets.

### Deviation from the original expression profile after probe filtering can be controlled by criterion on the overlapping length

**Figure 8:**
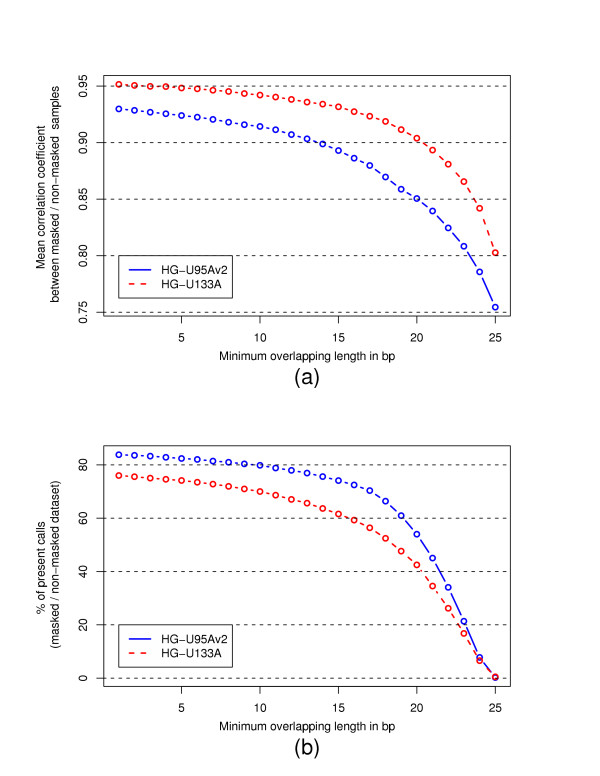
Deviation from the original expression profiles after probe filtering. (a) We compared the modified expression profile of each sample with the original one using the Spearman rank correlation coefficient. The mean correlation coefficient decreases as the minimum overlapping length increases. In addition, the effect of probe filtering is much stronger in U95Av2 than U133A. (b) Percentage of Present calls in the masked dataset compared to the original dataset. It drops down sharply after the minimum overlapping length of around 18 bp.

A reduction in the number of usable probes inevitably results in the deviation of the recomputed expression values from the original values calculated using all probes. Figure 8(a) shows the mean Spearman correlation coefficients between the expression values using all probes and those using only the selected probes by our criteria. We use the Spearman correlation here to capture the changes in the ranks of genes. As expected, the correlation decreases, as more stringent criteria are applied and a smaller subset of probes is chosen. Interestingly, the deviation in U95Av2 arrays is much larger than in U133A arrays, although the average fraction of used probes per probe set in each case is similar (see Figure 5 of Supplementary Material). For example, the mean correlation coefficient is greater than 0.9 in U133A when the criterion on the minimum overlapping length is less than 20 bp. For the same criterion, the mean correlation coefficient is about 0.85 in U95Av2. This appears to indicate that, in the process of making the two versions more similar, the larger changes occur to the expression levels in U95Av2 arrays. This result is consistent with the fact that probe design for U133A was performed in a more principled way than for U95Av2 and that U133A values are closer to the true values [25]. In addition to recalculating the expression values, the Affymetrix Present or Absent calls can also be calculated. Figure 8(b) shows the percentage of Present calls for each reduced group of probe sets. The probe filtering appears to reduce the percentage of Present calls, possibly because having fewer probes per probe set increases the likelihood of Absent calls. The usefulness of these calls can be debated; we simply present it here for those who find the calls helpful. In any case, we note that the percentage sharply drops down as the minimum overlapping length increases past 18 bp. Both Figures 8(a) and 8(b) indicate that 18–20 bp may be a reasonable cut-off values for the overlap length. We note that in filtering the probes, our goal is to simply make the expression profiles from U95Av2 and U133A more comparable. In the process, it is possible that this procedure sometimes results in less accurate expression values in absolute terms. By requiring that the probes in U133A have a sequence overlap with the less reliable set in U95Av2, we may be discarding some useful probes and, as a result, may be producing less accurate expression values. This is a trade-off that we make in order to utilize other data sets for a comparative study, but we should be aware of this fact in subsequent analysis.

## Conclusions

Comparative analysis of different microarray types has a potential to generate more comprehensive and reliable results by fully exploiting available data. Understanding and resolving both the inter-platform and inter-generation data remain an important and challenging practical issue. So far, attempts at such comparisons have been few, and many were limited to simple observations of low correlations in expression values. In this work, we provided a more quantitative and comprehensive description of the issues and inconsistencies through the analysis of a unique dataset consisting of HG-U95Av2 and HG-U133A hybridizations for each of the sample biopsies, and then we described a general method for resolving some of the problems.

We first observed in cluster analysis that with a standard matching of genes, the dominant feature of the dataset is not the sample characteristics but the array type. But we found that for clustering, this problem can be mitigated by rescaling each gene. We note, however, that this method is effective under certain assumptions, e.g., that there are enough samples for each array type and that each dataset does not contain unrelated experiments. If two groups of patients under study are measured on two different arrays, for example, a gene scaling will simply make the samples more homogeneous and reduce the differences between the groups. We also examined the inconsistencies in the list of differentially expressed genes obtained in the two cases. The overlap was very low, indicating that such a list may be platform-dependent and must be interpreted with caution. Some data filtering steps, either by selecting a subset of genes that are empirically shown to be well-correlated between platforms or by focusing only on highly-expressed genes, can be helpful at times, but they do not resolve the underlying problem.

Our approach based on the probe-level sequence information resulted in a significant improvement in the reproducibility in terms of correlation coefficients and selection of differentially expressed genes. As the probes aligned to multiple regions in the genome are eliminated and the probes that share larger segments are selected, the expression values become more consistent. This result is promising because it does not use data-dependent information such as the empirical correlation for each gene between different versions of arrays, which can only be obtained through special datasets such as ours. We examined the effect of the minimal sequence overlap length and the minimum number of probes per gene on the reproducibility, and found that, when the parameters are chosen properly, higher correlation can be attained while retaining a large number of probes for further analysis. We also examined the deviation from the original data when new expression values are calculated after probe filtering. In general, we recommend the minimum overlapping length of 18 ~ 20 bp and that at least 10 ~ 20% of probes in a probe set be present in the filtering step for a comparative analysis between U95Av2 and U133A.

Combining data across multiple platforms remains a formidable challenge. As a first step, we have studied the issues associated with combining data from multiple generations of a single platform and proposed one method. From our analysis, it is clear that technological issues can have significant effect and that one should be aware of the potential pitfalls in studies involving more than a single array type. In principle, the approach of selecting probes with sequence overlaps can be applied to other array types as well as to different versions of oligonucleotide arrays. For example, to study expression profiles of conserved regions across species using a different array for each species, more accurate results may be obtained by using only a subset of probes with sequence similarity. In each case, appropriate criteria for the length of overlap and the number of probes needed for a robust estimate of a probe set value need to be investigated for different contexts, but the results we provide in this work can serve as a guide.

## Methods

### Microarray data

Muscle tissue samples of 14 patients with inflammatory myopathies were collected. Among the 14 patients, 5 had dermatomyositis (DM) and 9 had other inflammatory myopathies including necrotizing myopathy, inclusion body myositis, granulomatous myositis, and polymyositis. Because the molecular profile of DM is sufficiently different from those of the rest, we can think of the DMs as one group and the rest as the other group in a two-group comparison [26]. Total RNA was extracted from muscle biopsy tissues and labeled. A portion was hybridized to HG-U95Av2 arrays; the remaining supply was frozen and then later hybridized to HG-U133A arrays at the same facility.

### Matching probe sets between U95Av2 and U133A

Although they belong to the same oligonucleotide array platform, the changes from the older version (U95Av2) to the newer one (U133A) were substantial: 1) Main source of probe selection region is different (UniGene Build 95 and 133; for the U133 set, other sequence databases such as dbEST were extensively used for choosing the probe selection region); 2) The number of probe pairs was reduced from 16 to 11 for a single gene; and 3) Probe selection method was improved [25]. The annotation for each probe set in U95Av2 and U133A was obtained from NetAffx Analysis Center (NetAffx annotation files (annotation date: 12/10/2003)) [27]. According to the annotation information, U95Av2 has 12,625 probe sets, which are annotated by 9,091 UniGene and 8,672 LocusLink identifiers. The newer version U133A consists of 22,283 probe sets annotated by 13,624 UniGene and 12,769 LocusLink identifiers. Here, the UniGene identifier was assigned by matching the representative sequence of each probe set to the UniGene database at the time of annotation. The LocusLink identifier was derived from the matched UniGene record (Annotation Methodology, Affymetrix web site).

For considering variations in the probe sets for the same transcript between different array versions, Affymetrix provides the probe set matching tables for comparative analysis. These matching tables were constructed based on the sequence information of probe sets as follows [21]. First, all possible probe set pairs between two array generations were checked by their similarity in the representative sequence for selection. Among the selected probe set pairs, "Good Match" pairs were chosen by the following criteria: 1) Percent identity between the representative sequences >90%; 2) Length of the representative sequence >100 base pairs (bp); 3) At least one perfect match (PM) probe of one array generation should be perfectly aligned to the probe selection region of the other array generation. In addition, "Best Match" is a subset of Good Match selected by more stringent criteria on the similarity of probe set pairs [21]. Best Match is used in the rest of the paper as it performs better than Good Match in all instances. When there is more than one probe set matching on either or both arrays, we take the average of the measurements.

### BLAT for the alignment of probes

For improving compatibility between U95Av2 and U133A, those probes whose sequence overlapped with any of the probes for the same gene on the other platform were selected. The extent of overlap necessary is described in the Results section. First, all the perfect match (PM) probes were aligned to the coding regions of the genome. Of commonly used short sequence alignment tools such as SIM4 [28], SPIDEY [29], and BLAT [30], we used BLAT (build version 26, available at  as a stand-alone program) because it appears to be more accurate and faster than others for matching short sequences with high sequence identity (more than 90%). BLAT has been used previously for annotating the probe sets of HG-U95Av2 in GeneAnnot system from Weizmann Institute of Science [31]. The alignment was done on the human chromosome sequence Build 34 (July 2003 freeze), available at UCSC Genome Bioinformatics ([32]). We ran BLAT with its default options (-tileSize = 11 -minMatch = 2 -minScore = 30, -minIdentity = 90 -maxGap = 2), without the overused tile file to avoid missing any matches. From the BLAT search result, only the 25-mer perfect alignments were considered for further analysis. All probes aligned to more than two regions in genomic DNA were discarded because of the possibility of cross hybridization. In each matched probe set pair, the overlapping lengths between all the possible PM probe pairings (16 × 11) were calculated.

### Filtering probes by overlapping length

The length of the overlap between probe sequences (1 bp ~ 25 bp) was used as a criterion for choosing probes for comparative analysis. The expression values were recomputed each time using only the selected probes by masking out the other probes from the raw (.cel) files. The values were calculated by the Statistical Expression Analysis Algorithm using Microarray Suite version 5.0 (MAS 5.0) (Affymetrix, Santa Clara, CA) without linear scaling to target intensity. MAS 5.0 is a robust estimator of expression index based on one-step biweight estimation algorithm, considering both perfect match (PM) and mismatch (MM) probes. This algorithm alleviates the problem of unstable expression values to some extent when a fraction of the probes is eliminated in our analysis.

## Authors' contributions

KBH carried out probe set matching, performed BLAT searches as well as statistical analysis, and drafted the manuscript. SWK carried out the raw data processing, performed statistical analysis, and provided input on drafts of the manuscript. SAG participated in the design of the study as well as providing the microarray data set for this study. PJP conceived the original idea of this study, participated in its design and coordination, and wrote sections of the manuscript. All authors read and approved the final manuscript.

## Supplementary Material

Additional File 1 Supplementary material for the paper "Combining gene expression data from different generations of oligonucleotide arrays" Supplementary figures for the paper Click here for file

## Figures and Tables

**Table 1:** Comparison of the methods for probe set matching. In the case of Best Match, the relation of probe sets between U95Av2 and U133A is many-to-one. The Pearson correlation coefficients of array pairs from the same biopsies were calculated and averaged for the assessment of comparability. The main reason for the high comparability of Best Match is the selection of the most appropriate probe set from the multiple matches using sequence information.

	No. of matched probe sets (U95Av2)	No. of matched probe sets (U133A)	No. of unique IDs shared between U95Av2 and U133A	Mean correlation coefficient of array pairs
UniGene IDs	11,596	15,858	8,867	0.832 ± 0.017
LocusLink IDs	11,389	15,666	8,661	0.831 ± 0.017
Best Match	10,507	9,530	9,530	**0.870** ± **0.016**
