# Supplementary material for: Combining gene expression data from different generations of oligonucleotide arrays
Source: BMC Bioinformatics. 2004 Oct 25;5:159. doi: 10.1186/1471-2105-5-159 (PMC528726; doi:10.1186/1471-2105-5-159)
Supplement: Additional File 1 — Supplementary material for the paper "Combining gene expression data from different generations of oligonucleotide arrays" Supplementary figures for the paper [file 1471-2105-5-159-S1.pdf]

Supplementary Material

Combining Gene Expression Data from Different Generations of Oligonucleotide Arrays

Kyu-Baek Hwang, Sek Won Kong, Steve A. Greenberg, Peter J. Park

June 29, 2004

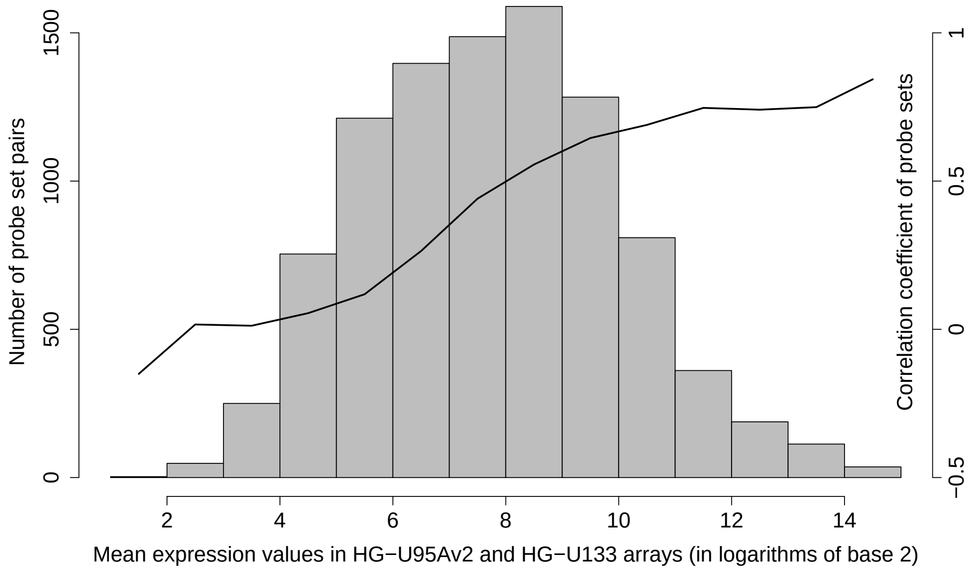

Figure 1: Relationship between average expression value and reproducibility. The histogram shows the distribution of the mean expression values for all genes (averaged across samples) in U95Av2 and U133A in the log scale. There are relatively few genes with very high or low expressions. For each interval, we calculate the correlation coefficient of each matched probe sets across the 14 array pairs and take the average of these correlations. The line indicates that this correlation coefficient increases for larger values of the mean expression level.

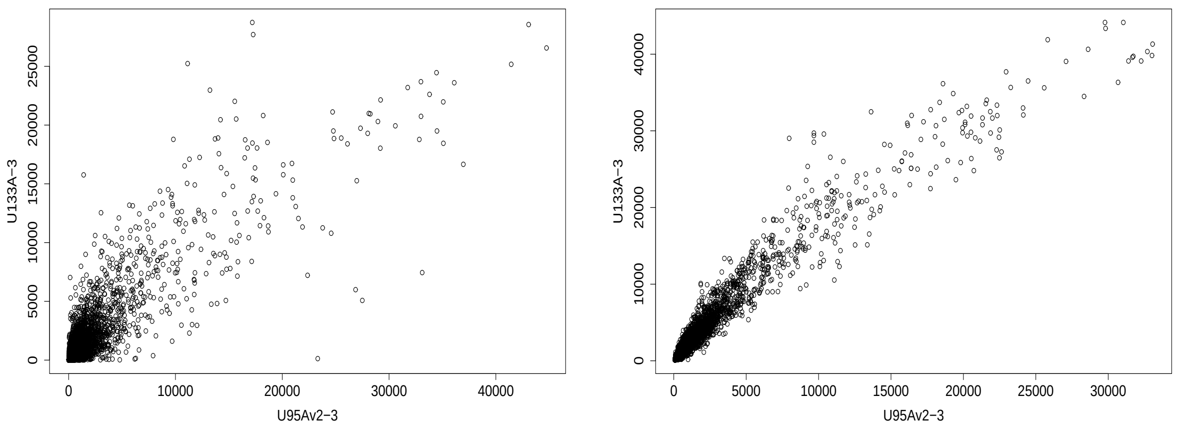

(a) (b)

Figure 2: Comparison of probe and probe set values between U95Av2 and U133A. (a) shows the values of *probe sets* matched by the Affymetrix Best Match scheme for Sample 3, displaying a large scatter between the arrays. This particular sample was chosen here because its correlation coefficient is closest to the mean of all the coefficients. (b) shows the PM values of the *probes* that have the same sequences in the two arrays, for the same sample. The number of probes having exactly same sequence across U95Av2 and U133A is 12,258. The reproducibility is much higher with the probes of same sequence (correlation coefficient of 0.964) than Best Match (correlation coefficient of 0.871).

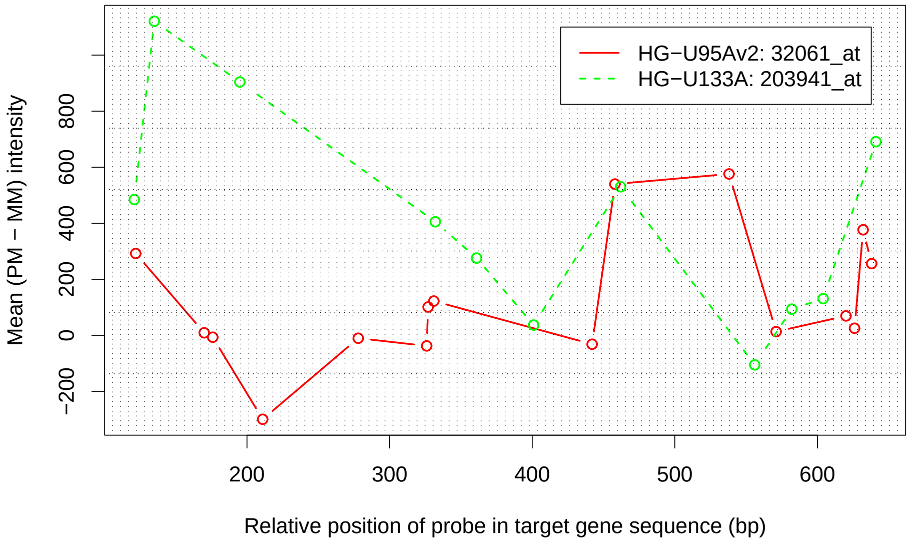

Figure 3: Intensity of each probe according to its position on the target gene sequence. The probe sets 32061_at in U95Av2 and 203941_at in U133A are a match according to Affymetrix Best Match. The intensity for each probe is an average across the 14 samples. Although all the probes are supposed to report the abundance of the same transcript, the probe-level intensities are very different depending on their position in the sequence. In the plot, differences in the intensity between overlapped probes are smaller than others. There are four matches between the probes of 32061_at and 203941_at with minimum overlapping length of 18 bp. The Pearson correlation coefficient between the expression patterns of 32061_at and 203941_at is about 0.386. Through probe filtering with minimum overlapping length of 18 bp, the correlation coefficient increases to about 0.865.

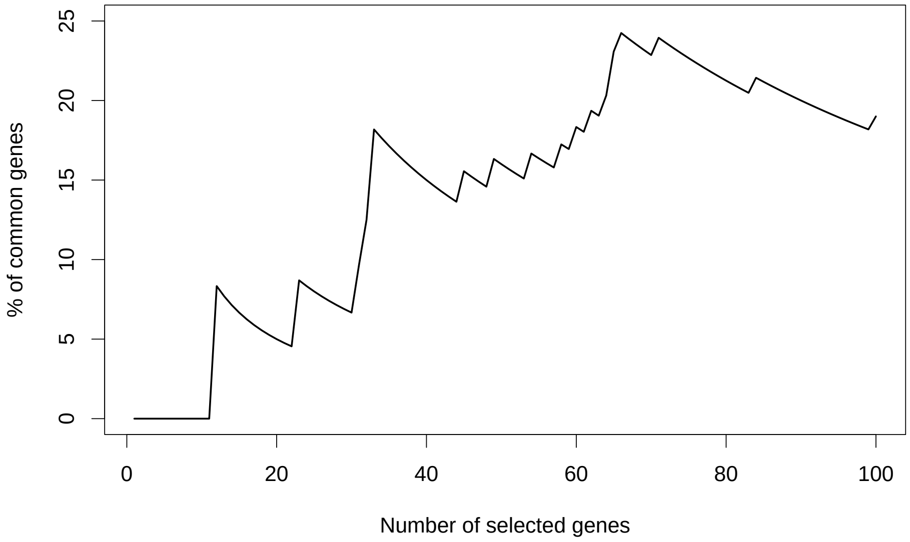

Figure 4: Percentage of differentially expressed genes common in U95Av2 and U133A datasets. The two datasets have a very small number of genes in common by the t-test. (For this analysis, the probe sets having more than 10 ‘Absent’ calls in either array generations were filtered out and the reminaing 5,381 genes were examined)

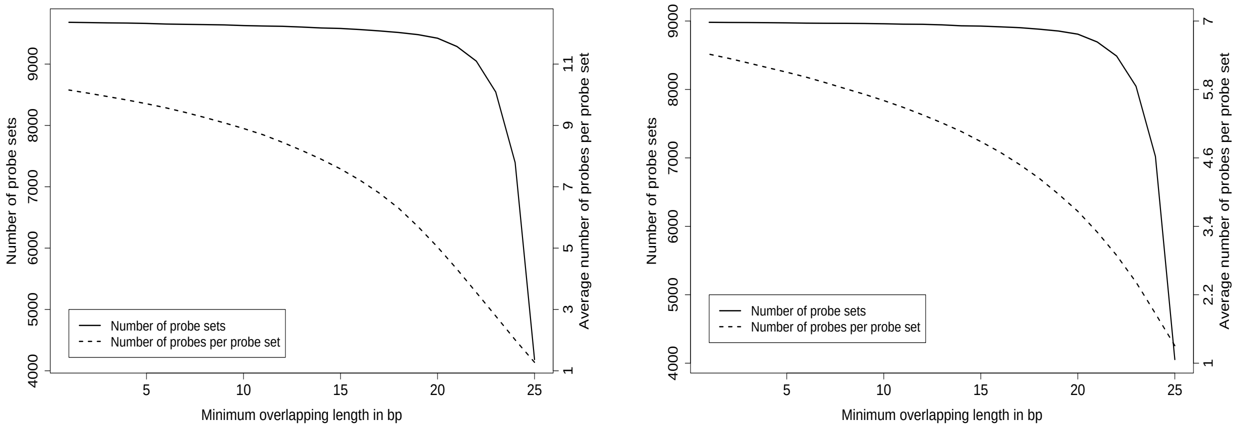

(a) U95Av2

(b) U133A

Figure 5: Variation in the number of usable probe sets (left y-axis) and the average number of eligible probes per such probe set (right y-axis) according to the criterion on minimum overlapping length. The usable probe set denotes the one which has at least one selected probe through probe filtering. The number of usable probe sets in each array platform does not vary substantially when the minimum overlapping length is less than 20 bp. After that, it decreases markedly as the criterion becomes more stringent. The average number of usable probes per such probe set decreases at a relatively constant rate as the minimum overlapping length increases.
